# Associations of estradiol levels and genetic polymorphisms of inflammatory genes with the risk of ischemic stroke

**DOI:** 10.1186/s12929-017-0332-1

**Published:** 2017-03-28

**Authors:** Yi-Chen Hsieh, Fang-I Hsieh, Yih-Ru Chen, Chaur-Jong Hu, Jiann-Shing Jeng, Sung-Chun Tang, Nai-Fang Chi, Huey-Juan Lin, Li-Ming Lien, Giia-Sheun Peng, Hung-Yi Chiou, for the Formosa Stroke Genetic Consortium (FSGC)

**Affiliations:** 1 Program for Neural Regenerative Medicine, College of Medical Science and Technology, Taipei Medical University, Taipei, Taiwan; 2 Center for Neurotrauma and Neuroregeneration, Taipei Medical University, Taipei, Taiwan; 3 Program in Biotechnology Research and Development, College of Pharmacy, Taipei Medical University, Taipe, Taiwan; 4 School of Public Health, College of Public Health, Taipei Medical University, Taipei, Taiwan; 5 Department of Neurology, Taipei Medical University Shuang Ho Hospital, New Taipei City, Taiwan; 6 Department of Neurology, School of Medicine, College of Medicine, Taipei Medical University, Taipei, Taiwan; 7 Cerebrovascular Research Center, Taipei Medical University, Taipei, Taiwan; 8 Stroke Center and Department of Neurology, National Taiwan University Hospital, Taipei, Taiwan; 9 Department of Emergency Medicine, Chi-Mei Medical Center, Tainan, Taiwan; 10 Department of Biotechnology, Southern Taiwan University of Science and Technology, Tainan, Taiwan; 11 Department of Neurology, Shin Kong Wu Ho-Su Memorial Hospital, Taipei, Taiwan; 12 School of Medicine, College of Medicine, Taipei Medical University, Taipei, Taiwan; 13 Division of Neurology, Department of Internal Medicine, Taipei Veterans General Hospital, Hsinchu Branch, Hsinchu County, Taiwan

**Keywords:** Ischemic stroke, Estradiol, Inflammation, Polygenic risk score

## Abstract

**Background:**

Estrogen plays an important role as an anti-inflammatory and neuroprotective agent in ischemic stroke. In this study, we analyzed the effect of a polygenic risk score (PRS) constructed using inflammatory genes and estradiol levels on the risk of ischemic stroke.

**Methods:**

This case-control study was conducted with 624 ischemic stroke patients and 624 age- and gender-matched controls. The PRS estimated the polygenic contribution of inflammatory genes from ischemic stroke susceptibility loci. Estradiol levels were measured using a radioimmunoassay. High and low estradiol levels were defined according to the log-transformed median estradiol levels in female and male controls.

**Results:**

Subjects in the fourth quartile of the PRS had a significant 1.57-fold risk of ischemic stroke (95% confidence interval [CI], 1.12 ~ 2.19), after adjusting for covariates compared to individuals in the lowest quartile. Compared to individuals with high estradiol levels and a low PRS as the reference group, those exposed to low estradiol levels and a high PRS had an increased risk of ischemic stroke (odds ratio, 3.35; 95% CI, 1.79 ~ 6.28). Similar results were also observed in males when the analysis was stratified by gender.

**Conclusions:**

Our data suggest that the PRS can be useful in evaluating a high risk of ischemic stroke among patients, especially those exposed to low estradiol levels.

## Background

Ischemic stroke accounts for 60% ~ 80% of all cerebrovascular diseases and is the third leading cause of death and the most common cause of permanent disability in developed countries and in Taiwan. Elucidation of the mechanisms involved in preventing cerebrovascular disease therefore remains an important research goal.

Neuroinflammation is a general process of several neurodegenerative diseases and may play an important role in the pathogenesis of ischemic stroke [1]. Inflammatory responses following cerebral ischemia underlie the important role of the innate immune system after an injury to the brain [2]. In a setting of cerebral injury, several transcription factors involved in the inflammatory response, including nuclear factor (NF)-κB, are activated, and subsequently proinflammatory genes, such as tumor necrosis factor (TNF)-α, monocyte chemotactic protein (MCP)-1, E-selectin, etc., are upregulated [3, 4]. Among them, MCP-1 is thought to play a pivotal role in the inflammatory process, since MCP-1 can combine with chemotactic cytokine receptor (CCR)-2 to activate macrophages and attract monocytes to the injured brain [5]. Afterwards, the cell adhesion molecule, E-selectin, increases in endothelial cells, damages the endothelial barrier, and regulates the attachment and transendothelial migration of leukocytes [6, 7].

Previous epidemiological studies also showed that estrogen exposure can postpone the onset of aging and reduce the risk of ischemic stroke and ischemic heart disease [8, 9]. In addition, a growing body of evidence suggests that estrogen has anti-inflammatory and neuroprotective functions [10]. In vivo and in vitro studies found that estradiol decreased expressions of MCP-1 and E-selectin [11, 12]. Several studies showed that decreases in estrogen-induced vascular inflammatory markers, including cytokines and adhesion molecules, might be the mechanism of vascular protection [13, 14]. Moreover, a novel and unique mechanism for the anti-inflammatory activity of 17β-estradiol (E_2_) was found, which indicated that E_2_ prevents inflammatory gene transcription induced by inflammatory agents by inhibiting NF-κB intracellular transport [15, 16].

Since estrogen can exert an antiatherosclerotic effect through modifying inflammatory genes, that might be a possible mechanism for its beneficial effect in cerebrovascular disease. In this study, we developed a polygenic risk score (PRS) of inflammatory genes for ischemic stroke and investigated the interaction between estradiol levels and the PRS of inflammatory genes on the risk of ischemic stroke.

## Methods

### Study design and participants

This case-control study was conducted with 624 ischemic stroke patients and 624 age- and gender-matched controls. Ischemic stroke patients who had suffered a first-ever episode of a symptomatic ischemic stroke and had been admitted to the department of neurology at National Taiwan University Hospital, Shuang Ho Hospital, Chi-Mei Medical Center, Shin Kong Wu Ho-Su Memorial Hospital, Tri-Service General Hospital, or Wanfang Hospital, Taiwan, were recruited as cases. Ischemic stroke was defined as a focal neurologic deficit of presumed vascular cause with sudden onset and lasting for at least 24 h as a recent infarct in a clinically relevant area of the brain on a computed tomographic (CT) or magnetic resonance imaging (MRI) brain scan performed within 10 days of the event. Controls were recruited from a community-based prospective study in Taipei which was described in a previous study [17]. Some of the control subjects were selected from those who attended an annual health examination at the health center at Taipei Medical University Hospital. After frequency matching by age and gender with cases and excluding those with a history of stroke or heart disease, 624 subjects were enrolled as controls. In addition, participants were excluded if they were taking hormone-related drugs. The study was approved by the ethics committees of the participating hospitals and Taipei Medical University with the understanding that all data would be coded and patient anonymity was guaranteed. Each subject provided written informed consent prior to participation in the study.

### Data collection and risk factor definition

Information pertaining to the age, gender, and presence of major vascular risk factors was collected from patients and controls using a structured questionnaire. The height, weight, waist and hip circumferences, total cholesterol, triglyceride, high-density lipoprotein cholesterol (HDL-C), low-density lipoprotein cholesterol (LDL-C), and fasting glucose were measured. The body-mass index (BMI) was defined as an individual’s body weight (kg) divided by the square of their height (m^2^). The waist-to-hip ratio (WHR) was computed using the index of waist circumference divided by the hip circumference. Subjects who had ever smoked at least 100 cigarettes during their entire life were defined as a cigarette smoker, while subjects who had drunk alcohol at least once per day during a year were categorized as an alcohol drinker. These two factors were then combined for analysis in the study. The above-mentioned information was obtained in an interview with study subjects and a review of medical charts by trained research assistants/nurses. Hypertension was defined as systolic blood pressure of >140 mmHg or diastolic blood pressure of >90 mmHg or the use of antihypertensive drugs. Diabetes was defined as fasting glucose of ≥126 mg/dL or the use of pharmacological treatment.

### Estradiol analyses

The laboratory assay for estradiol was a radioimmunoassay. The lower limit of quantitation was 2 pg/ml for estradiol. Duplicate samples were included for 10% of subjects for quality control purposes, and the coefficient of variation was 8%. Samples were labeled in such a way that laboratory personnel were unaware of the case-control status of the samples or the identity of the duplicates. In this study, although estradiol levels were measured for only 513 ischemic stroke patients and 383 healthy controls due to a limited research budget, characteristics of study subjects did not differ between those whose estradiol level was measured and those for whom it was not. Additionally, estradiol concentrations were log-transformed to reduce departures from a normal distribution. High and low estradiol levels were defined according to median log-transformed estradiol levels in premenopausal or postmenopausal female and male controls.

### DNA extraction, single-nucleotide polymorphism (SNP) selection, and genotyping

Genomic DNA was extracted from EDTA-anticoagulated peripheral blood according to the phenol chloroform protocol and stored at -80 °C until used for further analysis. For the selection of SNPs, Haploview software was used to conduct linkage disequilibrium and haplotype block analyses, using the Hapmap phase II genotype data for inflammatory genes including MCP-1, CCR2, and E-selectin in data of Han Chinese from Beijing, China. Selection of tagSNPs was performed by running the tagger program implemented in both the Haploview [18] and tagSNPs programs [19]. The criteria for *r* ^2^ was set to >0.8. Another way to choose SNPs as tagSNPs was based on SNPs located in the promoter region which might well plausibly have a functional role. SNP genotyping was performed using a polymerase chain reaction-restriction fragment length polymorphism (PCR-RFLP) method. To assure the genotyping quality, a detailed quality control procedure including the duplicate identification of genotypes, a Hardy-Weinberg Equilibrium (HWE) test, and a call rate of >99% were measured. In total, 55 (5%) duplicate samples were successfully genotyped, and the concordance rate was 100%.

### Polygenic risk score (PRS)

A PRS was used to evaluate polygenic susceptibility loci which contributed to ischemic stroke, and was established according to SNPs under a specified *p*-value threshold (*p* < 0.15) on any one of the per-allele dominant or recessive logistic regression models [20], which indicated a significant main effect of polygenic risk for ischemic stroke. When there was strong linkage disequilibrium between SNPs located on the same gene, the variant with the lowest *p* value was selected as the candidate. The total of the product of the number of risk allele copies of candidate SNPs and the corresponding log odds estimate were subsequently measured as a weighted PRS. A multivariate logistic regression model including the PRS, age, BMI, hypertension, diabetes mellitus, heart disease, and stroke family histories was utilized to explore the independent association of the PRS with the risk of ischemic stroke. Use of a bootstrap method with 1000 repetitions to adjust model regression coefficients was to prevent a potential overfitting issue. In this procedure, bootstrapping was repeatedly and randomly resampled by extracting samples with replacement from the original dataset. Then cross-validation and split-sample procedures were applied to enhance the accuracy of the prediction for internal validation [21, 22]. In this step, the dataset was assigned to k subsets, which formed the training set, and the holdout method was repeated k times. The other k-1 subsets were merged to constitute a training set. Moreover, we partitioned the original dataset into training and validation samples, including 80% and 20% of the original dataset, respectively.

### Statistical analysis

Student’s *t*-test was used to compare continuous variables between cases and controls. The Chi-squared test was applied to test differences in categorical variables. A multivariate logistic regression model carried out with adjustment for covariates, which significantly differed between cases and controls in demographic and clinical characteristics, was used to estimate odds ratios (ORs) and 95% confidence intervals (CIs). HWE for each polymorphism was tested in the controls. Multiple testing correlation was performed using the permutation test. An additive effect of the estradiol level and PRS were estimated using the relative excess risk resulting from interaction (RERI). The power estimation of this gene-environment interaction study was determined using the QUANTO program [23]. All analyses were performed with SAS Genetics software (vers. 9.4; SAS Institute, Cary, NC, USA). Two-tailed *p* values were calculated, and statistical significance was set to *p* < 0.05.

## Results

### Baseline characteristics of study subjects

Basic demographic and clinical characteristics of study subjects are shown in Table 1. Mean ages of subjects were 65.6 ± 10.8 years for cases and 65.0 ± 10.5 years for controls. Ischemic stroke patients were significantly more obese than healthy controls. The frequencies of either one or both cigarette smoking/alcohol consumption habits were significantly higher in patients than in controls. Prominently higher percentages of hypertension, diabetes mellitus, heart disease, and family stroke history were observed for cases than for controls. The distribution of estradiol levels in patients was significantly higher than in healthy subjects for both females and males.

**Table 1:** Demographic and clinical characteristics of the study population

	Ischemic stroke patients		Healthy control		*p* value
	*N* = 624		*N* = 624		
Age (years)	65.6 ± 10.8		65.0 ± 10.5		---
Gender					
Female	212	(34.0)	212	(34.0)	---
Male	412	(66.0)	412	(66.0)	
Body-mass index (kg/m^2^)	25.0 ± 3.9		24.6 ± 3.1		0.0212
Waist-hip ratio	0.94 ± 0.08		0.87 ± 0.07		<0.0001
Cigarette smoking/alcohol consumption					
Never/Never	296	(47.6)	408	(65.8)	<0.0001
Either one	203	(32.6)	177	(28.6)	
Ever/Ever	123	(19.8)	35	(5.7)	
Hypertension	482	(77.2)	371	(59.5)	<0.0001
Diabetes mellitus	300	(48.1)	105	(16.8)	<0.0001
Heart disease	163	(26.1)	109	(17.5)	0.0002
Dyslipidemia	476	(76.5)	504	(80.8)	0.0677
Family stroke history	149	(23.9)	97	(15.5)	0.0008
Estradiol level^a^					
Female	155	2.90 (0.97)	128	1.68 (1.36)	<0.0001
Male	358	3.18 (0.54)	255	2.97 (0.57)	<0.0001

Data are presented as *n* (%) or mean ± standard deviation

^a^Estradiol level was shown in log transformed value

### Association between inflammatory genes and ischemic stroke risk

Genotype frequencies of MCP-1 (rs1024611 and rs3760396), CCR2 (rs1799864 and rs1799865), and E-selectin (rs2076059, rs10800469, rs3917412, and rs5368) are depicted in Table 2. These SNPs were all under HWE. There were no prominent associations between SNPs on MCP-1, CCR2, or E-selectin genes in addition to rs2076059, which was located on E-selectin and significantly increased the risk of ischemic stroke under the dominant model (Table 2).

**Table 2:** Association between inflammatory genes and the risk of ischemic stroke

Gene	SNP ID	Allele risk/reference	OR^a^ (95% CI)					
			Per-allele	*p* value	Dominant model	*p* value	Recessive model	*p* value
MCP-1	rs1024611	A/G	1.16(0.96–1.39)	0.1154^b^	1.23(0.93–1.63)	0.1492	1.19(0.87–1.64)	0.2753
	rs3760396	C/G	0.93(0.69–1.27)	0.6585	1.02(0.73–1.43)	0.9120	0.29(0.09–1.26)	0.1420
CCR2	rs1799864	C/T	1.14(0.89–1.47)	0.3062	1.36(0.67–2.74)	0.3922	1.15(0.84–1.56)	0.3851
	rs1799865	C/T	1.06(0.87–1.29)	0.5615	1.00(0.77–1.31)	0.9882	1.27(0.85–1.91)	0.1424^b^
E-selectin	rs2076059	T/C	1.26(0.96–1.66)	0.0950	1.37(1.01–1.84)	0.0411^b^	0.73(0.26–2.08)	0.5605
	rs10800469	A/G	1.02(0.86–1.22)	0.8060	0.97(0.74–1.28)	0.8266	1.11(0.85–1.50)	0.5074
	rs3917412	A/G	1.00(0.82–1.21)	0.9769	0.92(0.71–1.19)	0.5150	1.28(0.84–1.94)	0.2553
	rs5368	T/C	1.11(0.90–1.38)	0.3368	0.78(0.42–1.42)	0.4118	1.22(0.94–1.58)	0.1330^b^

*Abbreviations*: *OR* odds ratio, *CI* confidence interval

^a^ORs were adjusted for age, body-mass index, cigarette smoking/alcohol consumption, hypertension, diabetes mellitus, heart disease, and stroke family history

^b^Included in the polygenic risk score

### Association between PRS and ischemic stroke risk

Concerning development of the PRS, four SNPs, namely rs1024611, rs1799865, rs2076059, and rs5368, were selected to create the PRS model. Since two SNPs located on MCP-1 were in strong linkage disequilibrium (D’ > 0.9) and both reached the criteria to be chosen as candidate SNPs for the PRS, rs1024611 was picked due to its lower *p* value. We choose two SNP, rs2076059 and rs5368, on the same E-selectin gene because of low linkage disequilibrium (D’ < 0.6) (Table 2).

Based on the quartile distribution of healthy controls, subjects in the second (0.2033 ~ 0.3634), third (0.3635 ~ 0.4933), and fourth quartiles (>0.4933) of the PRS had 1.12-, 1.17-, and 1.57-fold risks of ischemic stroke, respectively, after adjusting for covariates compared to individuals in the lowest quartile (Table 3).

**Table 3:** Association between the polygenic risk score and the risk of ischemic stroke

Polygenic risk score quartiles	Total		
	Cases/Controls	OR^a^ (95% CI)	*P*-value
1st	254/300	1.0	
2nd	112/114	1.12(0.75–1.65)	0.5801
3rd	74/77	1.17(0.75–1.84)	0.5029
4th	184/133	1.57(1.12–2.19)	0.0084
		*p* for trend	0.0050

^a^Adjusted for age, body mass index, cigarette smoking/alcohol consumption, hypertension, diabetes mellitus, heart disease, and stroke family history

### Effects of estradiol level and PRS on ischemic stroke risk

**Fig. 1:**
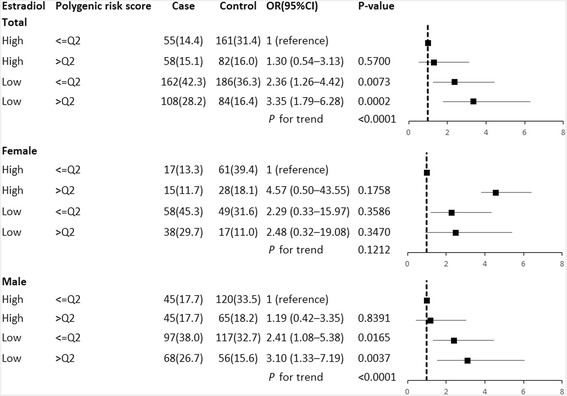
Combined effect of estradiol levels and polygenic risk score on the risk of ischemic stroke. The odds ratios (ORs) were adjusted for age, cigarette smoking/alcohol consumption, hypertension, diabetes mellitus, heart disease, and stroke family history. Estradiol levels were divided into high and low groups according to median log-transformed estradiol levels in female and male controls. The second quartile (Q2) of the polygenic risk score in controls was used as a cutoff point

Figure 1 indicates the effect of estradiol levels and the PRS on the risk of ischemic stroke stratified by gender. Relative to people with high estradiol levels and a lower PRS as the reference group, those with either risk factor had increased risk of ischemic stroke, while subjects exposed to a low estradiol level and a higher PRS had a significant 3.35-fold risk of ischemic stroke (95% CI, 1.79 ~ 6.28, *p* = 0.0002). An additive interaction effect was observed; however the statistic did not reach significance due to the small sample size (RERI = 0.36; 95% CI, -0.96 ~ 1.68). A similar tendency was observed for males when the analysis was stratified by gender.

## Discussion

In this study, we attempted to investigate the interaction between estradiol levels and inflammatory genes including MCP-1, CCR2, and E-selectin on the risk of ischemic stroke. The PRS was developed and included four of eight SNPs examined. A higher score indicated a higher risk of ischemic stroke. The risk was exacerbated when a subject was exposed to a low estradiol level and had a higher PRS.

MCP-1, which is located on chromosome 17q11-21 and is composed of three exons and two introns, belongs to the chemotactic cytokine family. MCP-1 -2518A > G (rs1024611) and -927G > C (rs3760396) situated in the distal promoter regulatory region of the MCP-1 gene could affect the transcription of MCP-1 [24]. Two recent meta-analysis studies suggested that MCP-1 rs1024611 is associated with ischemic heart disease and ischemic stroke susceptibilities [25, 26]. Furthermore, cerebral infarction patients had higher levels of serum MCP-1 than did healthy control subjects; however, two SNPs, rs1024611 and rs3760396, were not remarkably associated with ischemic stroke, which might have been due to ethnic differences since most significant findings were observed among Caucasian populations [26].

CCR2, resident on chromosome 3q21, transcribes the receptor of MCP-1 which plays important roles in extravasation and transmigration of monocytes under inflammatory conditions [5, 27]. There were no nominal associations of CCR2 rs1799864 or rs1799865 with the risk of ischemic stroke in our study. This result is consistent with previous studies conducted in an Armenian population but not in a Chinese Han population, since a significant association was observed between CCR2 and ischemic stroke only in the hypertensive group [28, 29].

The E-selectin gene, a member of the selectin superfamily of adhesion molecules, is on chromosome 1q23-25. In our study, E-selectin rs2076059 was significantly associated with ischemic stroke under the dominant model after adjusting for covariates. E-selectin rs2076059 is in a non-coding region. There is no doubt that polymorphisms located in exon regions have higher probabilities of having biological functions. However, we could not exclude the possibility that polymorphisms in non-expression regions might interfere with gene expressions, since polymorphisms situated in an enhancer region consisting of coded or uncoded sequences physically affect regulation of gene expressions.

Because stroke is a complicated disease which is not attributed to a single gene mutation, a PRS estimated by a sum of trait-associated alleles across many genetic loci and weighted by an effect size is more appropriate. A PRS constructed according to the magnitudes of individual SNPs' effects would be most efficient when considering multiple loci in combination [30, 31]. In this study, MCP-1, its receptor, CCR2, and E-selectin which are prominently influenced by E2 were selected as candidate genes. The PRS construction procedure of these three candidate genes included eight SNPs, and MCP-1 rs1024611, CCR2 rs1799865, and E-selectin rs2076059 and rs5368 were chosen to build a PRS. Our findings that the risk of developing a specific inflammation-related disease might be influenced by a susceptibility profile are consistent with previous studies, and reflect the combined effects of multiple high-risk alleles [32, 33]. A PRS model comprised of five variants in a Japanese study showed that the OR for ischemic stroke was 1.75-fold in the highest PRS quintile compared to the lowest [32]. Opherk et al. further found a PRS to be associated with white matter hyperintensity volume in cerebral small-vessel disease [33]. In short, these findings suggest that a PRS can provide valuable information for individual risk assessments.

Estrogen is considered to have well-documented neuroprotective effects in a variety of experimental and clinical neurodegenerative disorders including stroke [34, 35]. A mechanism to explain the anti-inflammatory activity of E_2_ is through estrogen receptor-α (ERα), which mediates inhibition of NF-κB transport [15]. Further, E_2_ can reduce MCP-1 and E-selectin expressions in cell culture preparations [15, 36], in experimental animals [37, 11], and even in human studies [38]. Since estrogen has important gene-regulatory effects [39], it was not surprising to find that subjects with a low level of estradiol and a higher PRS had higher ischemic stroke risks.

Our data have some valuable strengths. First, this is the first study to simultaneously investigate the effects of estrogen and inflammatory genetic polymorphisms on the risk of ischemic stroke. Second, the diagnostic criteria of ischemic stroke were quite accurate since all patients were confirmed by CT or MRI. However, this study has several potential limitations. First, this was a case-control study design, and recruitment and survival bias could not be neglected. Second, although this study had nearly 80% power to detect a log-additive model with an OR of 1.5, allelic frequencies of 10%, and an interaction effect size of R_ge_ = 2, other SNPs with ORs of <1.5 may need a larger sample size to increase the statistical power. Therefore, further studies with larger sample sizes are warranted. Third, only SNPs of MCP-1, CCR2, and E-selectin genes were selected to investigate the relationship with ischemic stroke. Other SNPs of inflammatory genes might be associated with ischemic stroke and must be further studied to clarify the role of inflammatory genes and estrogen in ischemic stroke. Last, although around 90% subjects were postmenopausal, we could still measure the estradiol level especially in females and found the effect of the hormone on the risk of ischemic stroke.

## Conclusions

Our results show that the PRS model composed of four variants of inflammatory genes could identify those with a high risk of ischemic stroke. Furthermore, the risk worsened when subjects were exposed to low estradiol levels. Future comprehensive evaluations of the PRS with estradiol levels and additional lifestyle or environmental risk factors should be considered.

## Abbreviations

BMI: Body-mass index
CCR: Chemotactic cytokine receptor
CI: Confidence interval
CT: Computed tomography
E_2_: 17β-estradiol
ERα: Estrogen receptor-α
HDL-C: High-density lipoprotein cholesterol
HWE: Hardy-Weinberg equilibrium
LDL-C: Low-density lipoprotein cholesterol
MCP-1: Monocyte chemotactic protein-1
MRI: Magnetic resonance imaging
NF-κB: Nuclear factor-κB
OR: Odds ratio
PCR-RFLP: Polymerase chain reaction-restriction fragment length polymorphism
PRS: Polygenic risk score
RERI: Relative excess risk resulting from interaction
SNP: Single-nucleotide polymorphism
TNF-α: Tumor necrosis factor-α
WHR: Waist-to-hip ratio
